# Risk of Dementia in Patients with Insomnia and Long-term Use of Hypnotics: A Population-based Retrospective Cohort Study

**DOI:** 10.1371/journal.pone.0049113

**Published:** 2012-11-07

**Authors:** Pin-Liang Chen, Wei-Ju Lee, Wei-Zen Sun, Yen-Jen Oyang, Jong-Ling Fuh

**Affiliations:** 1 Department of Computer Science and Information Engineering, National Taiwan University, Taipei, Taiwan; 2 Department of Neurology, Taichung Veterans General Hospital, Taichung, Taiwan; 3 Faculty of Medicine, National Yang-Ming University School of Medicine, Taipei, Taiwan; 4 Institute of Clinical Medicine, National Yang-Ming University, Taipei, Taiwan; 5 Department of Anesthesiology, National Taiwan University Hospital, Taipei, Taiwan; 6 Department of Neurology, Neurological Institute, Taipei Veterans General Hospital, Taipei, Taiwan; 7 Graduate Institutes of Biomedical Electronics and Bioinformatics, National Taiwan University, Taipei, Taiwan; “Mario Negri” Institute for Pharmacological Research, Italy

## Abstract

**Background:**

Hypnotics have been reported to be associated with dementia. However, the relationship between insomnia, hypnotics and dementia is still controversial. We sought to examine the risk of dementia in patients with long-term insomnia and the contribution of hypnotics.

**Methods:**

Data was collected from Taiwan’s Longitudinal Health Insurance Database. The study cohort comprised all patients aged 50 years or older with a first diagnosis of insomnia from 2002 to 2007. The comparison cohort consisted of randomly selected patients matched by age and gender. Each patient was individually tracked for 3 years from their insomnia index date to identify whether the patient had a first diagnosis of dementia. Cox regression was used to estimate hazard ratios (HRs) and 95% confidence intervals (CIs).

**Results:**

We identified 5693 subjects with long-term insomnia and 28,465 individuals without. After adjusting for hypertension, diabetes mellitus, hyperlipidemia, and stroke, those with long-term insomnia had significantly higher risks of dementia (HR, 2.34; 95% CI, 1.92–2.85). Patients with long-term insomnia and aged 50 to 65 years had a higher increased risk of dementia (HR, 5.22; 95% CI, 2.62–10.41) than those older than 65 years (HR, 2.33; 95% CI, 1.90–2.88). The use of hypnotics with a longer half-life and at a higher prescribed dose predicted a greater increased risk of dementia.

**Conclusions:**

Patients with long-term use of hypnotics have more than a 2-fold increased risk of dementia, especially those aged 50 to 65 years. In addition, the dosage and half-lives of the hypnotics used should be considered, because greater exposure to these medications leads to a higher risk of developing dementia.

## Introduction

Hypnotics are among the most frequently used drugs for patients with insomnia. They can be classified as benzodiazepines (BZDs) and non-benzodiazepines (non-BZDs). Some studies have reported that using BZDs is associated with dementia, but the mechanisms are not clear, and there are fewer studies on non-BZDs and dementia. Fastbom et al. reported a significantly lower incidence of dementia in elderly persons using BZDs [1]. On the other hand, other studies reported that the use of BZDs is associated with a significantly increased risk of dementia [2], [3]. In addition, Wu et al. suggested that the risk of dementia, which was high for current BZD users, decreased as the duration of BZD discontinuation lengthened [3]; Lagnaoui et al. reported the opposite findings [2].

Patients with Alzheimer's disease (AD) who were taking antipsychotic drugs and those taking hypnotics were more likely to have a faster rate of deterioration than those who were not taking any of these drugs [4]. In addition to BZDs, many studies reported that severe mental illness (SMI) was associated with dementia. Depression was associated with an increased risk of dementia and AD [5], [6], [7], [8], [9], [10], [11], [12], [13], [14]. The risk of dementia increased with the number of episodes of bipolar affective disorders [10]. Anxiety symptom was not associated with dementia and AD [15], [16].

Previous studies have provided no comprehensive analysis of the relationship between insomnia and hypnotics and dementia because of limited subject numbers and the use of biased populations (such as hospital-based cohorts). In order to include adequate cases and acquire a robust estimation of the potential role of insomnia, age, sex, and hypnotics in dementia, we required data from a large representative population that had been followed up for a sufficient length of time. The primary goal of the present study was to examine the risk of dementia between patients with and without insomnia using a large population-based database from Taiwan. The secondary goal was to examine the possible association between hypnotics use and the risk of dementia in the elderly.

## Methods

### Study Population

This study used the Longitudinal Health Insurance Database (LHID) derived from Taiwan’s National Health Insurance Research Database (NHIRD). The NHIRD covered 23 million registered patients from March 1995 to December 2010, representing more than 99% of the entire population of Taiwan. Data for the LHID was collected by systematically and randomly sampling from the NHIRD; the database included the data of 1 million individuals. The National Health Research Institute of Taiwan reports that there were no significant differences in gender distribution, age distribution, or average insured payroll-related amount between the patients in the LHID and those in the original NHIRD [17].

Several studies have used the NHIRD to find associations between different diseases [18], [19], [20]. Cheng et al. reported that the accuracy of the NHIRD in recording ischemic stroke diagnoses and aspirin prescriptions was high, and that the NHIRD appears to be a valid resource for population research in ischemic stroke [21].

### Study Sample

In our retrospective cohort study, the study cohort comprised all patients who were older than 50 years with a first diagnosis of insomnia (International Classification of Disease [ICD]-9-CM code 780.52) from January 1, 2002 to December 31, 2007. To ensure that all patients had long-term insomnia, we included only those patients who had been diagnosed with insomnia twice within one year and been prescribed at least 30 defined daily doses (DDD) of hypnotics per year, to be used at night. Since SMI has been associated with an increased risk of dementia, we excluded all patients with a diagnosis of any SMI from 2000 to 2010, including neurotic depression, depressive disorder, anxiety states, anxiety disorder, bipolar disorder and schizophrenia, and to avoid the influence of Parkinson’s disease (PD), we also excluded all patients with a PD diagnosis. In addition, we randomly identified 5 controls for each insomnia subject. First, we looked for those who were of the same age and sex as each insomnia subject in our database. Second, we excluded patients with an insomnia diagnosis, SMI diagnosis, PD diagnosis or use of hypnotics. Third, we set a time seed and used the random function in Perl to choose 5 controls randomly. If a person were chosen, he/she would not be chosen again.

We examined the risk of dementia, including pre-senile dementia and senile dementia (ICD-9-CM code 290.0–290.3) and AD (ICD-9-CM code 331.0). Arteriosclerotic dementia (ICD-9-CM code 290.4) was excluded. Each patient was individually tracked for 3 years from their insomnia index date to identify whether the patient had a first diagnosis of dementia during the follow-up period.

### Covariates

We extracted demographic information, including age and sex, and possible confounding factors, including hypertension, type 2 diabetes mellitus, hyperlipidemia, and stroke. Insomnia patients and controls were coded as having a confounding factor if there was a recorded diagnosis before their dementia index date.

To assess the differences in the risks of dementia in terms of different covariates, we divided the sample into 2 groups based on age (those between 50 and 65 years and those 65 or more). Hypnotics were classified into 2 groups: BZDs and non-BZDs. The BZDs included nordazepam, clonazepam, flurazepam, etc. The non-BZDs included zopiclone, zolpidem and zaleplon. Other drugs such as trazodone or melatonin agonists are not commonly used for insomnia in Taiwan. In addition, we classified hypnotics into 3 groups according to their half-life values, as defined by the World Health Organization [22], [23]: short-acting (less than 5 hours), intermediate-acting (from 5 to 24 hours), and long-acting (more than 24 hours). We classified a patient into one of the 3 groups based on whether the patient had been prescribed at least 30 DDD of a drug in one of the drug groups per year. If a patient used both BZDs and non-BZDs, the patient would be excluded from the analysis of BZDs and non-BZDs. However, if a patient switched from shorter-acting to longer-acting hypnotics, the patient would be moved into the longer-acting group. For example, if a patient received zolpidem and nordazepam, the patient was excluded when performing analysis of BZDs or non-BZDs. However, the patient was placed into the long-acting hypnotics group.

We counted the dosages of hypnotic prescriptions during each subject’s follow-up period, and the cumulative dose of hypnotics was transferred into DDD. To analyze the contribution of the hypnotic dosages more precisely, we included additional subjects who had been prescribed hypnotics at between 7 and 30 DDD per year. Subjects were categorized as low-dose users if they had been prescribed 7 to 30 DDD per year, medium-dose users if they had been prescribed 31 to 90 DDD per year, and high-dose users if they had been prescribed at least 91 DDD per year.

Subjects with exposure to any hypnotics during the study period were identified. Subjects were defined as current users if their prescription of hypnotics ended within 30 days before their respective dementia index date; previous users if their prescription ended 31 to 90 days before their dementia index date; and remote users if their prescription ended 91 days or more before their dementia index date.

### Statistical Analysis

All statistical procedures were performed with the statistical software package SAS for Windows (version 9.2.; SAS Institute Inc., Cary, NC, USA). The clinical variables were compared between cases and controls using the chi-square test. Cox regression was used to estimate hazard ratios (HRs) and 95% confidence intervals (CIs). We adjusted the models for the possible confounding factors of hypertension, type 2 diabetes mellitus, hyperlipidemia, and stroke. All tests were 2-tailed, and *p* values <0.05 were considered significant.

## Results

We identified 7957 subjects with long-term insomnia, of which 2264 were excluded because of a diagnosis of SMI or PD, leaving 5693 subjects for final analysis. The control group without insomnia comprised 28,465 subjects. The prevalence of comorbid illness, including hypertension, diabetes mellitus, hyperlipidemia and stroke, was higher for the long-term insomnia subjects than for the control group (*p*<0.001). Of the 5693 long-term insomnia subjects, more had been prescribed non-BZD (49.4%) than BZD drugs (33.6%). Nine hundred sixty-six subjects (17.0%) had been prescribed BZDs and non-BZDs at the same time; 273 patients (5.76%) switched from short-acting to intermediate-acting or long-acting hypnotics and 51 (5.39%) switched from intermediate-acting to long-acting hypnotics. Short-acting hypnotics were the most commonly prescribed (78.4%). Almost half the patients (47.8%) were prescribed at least 91 DDD per year. **Table 1** and **Table 2** reveal the demographic characteristics of subjects with and without hypnotic usage in the study.

**Table 1:** Comparison of subjects with and without hypnotic use*.

Characteristic	Hypnotic Users (N = 5693)		Hypnotic Nonusers(N = 28,465)		*P* Value+
Age, median (IQR), y	65	(57–74)	65	(57–74)	1.000
50–65	2785	(48.9%)	13,925	(48.9%)	
>65	2908	(51.1%)	14,540	(51.1%)	
Sex					1.000
Male	2520	(44.3%)	12,600	(44.3%)	
Female	3173	(55.7%)	15,865	(55.7%)	
Comorbidities					
Hypertension	3163	(55.6%)	10,483	(36.8%)	<.001
Diabetes	1456	(25.6%)	4904	(17.2%)	<.001
Hyperlipidemia	1907	(33.5%)	6092	(21.4%)	<.001
Stroke	1161	(20.4%)	3522	(12.4%)	<.001

Abbreviation: IQR, interquartile range.

* Data are number (percentage) except where indicated.

+Group comparisons by the chi-square test.

**Table 2:** Characteristics of subjects with insomnia and hypnotic use (N = 5693).

Group	No. (%)	
Category		
BZD	1915	(33.6)
non-BZD	2812	(49.4)
BZD and non-BZD	966	(17.0)
Half-life#		
Short-acting	4465	(78.4)
Intermediate-acting	895	(15.7)
Long-acting	333	(5.8)
Dose*		
Low$	2718	
Medium	2971	(52.2)
High	2722	(47.8)
Period+		
Remote	3434	(60.3)
Previous	792	(13.9)
Current	1467	(25.8)

Abbreviation: BZD, benzodiazepine.

# There were 273 patients (5.76%) switched from short-acting to intermediate-acting (N = 199) or long-acting (N = 74) and 51 patients (5.39%) switched from intermediate-acting to long-acting hypnotics.

* Low: 7 to 30 defined daily dose (DDD) per year; medium: 31 to 90 DDD per year; high: at least 91 DDD per year.

$ Additional included subjects.

+ Current: prescription of hypnotics ended within 30 days before dementia index date; previous: prescription ended 31 to 90 days before dementia index date; remote: prescription ended 91 days or more before dementia index date.

During the following 3 years, 220 of the 5693 subjects (3.86%) with hypnotic usage were diagnosed with dementia. Those with hypnotic usage had a significantly higher risk of dementia than those without hypnotic usage (*p*<0.001; HR, 2.34; 95% CI, 1.92–2.85). Both male patients (HR, 2.28; 95% CI, 1.68–3.10) and female patients (HR, 2.39; 95% CI, 1.85–3.09) with hypnotic usage had higher risks of dementia, but there were no significant differences between them. Patients older than 65 years had a higher risk of dementia than those between 50 and 65 years of age. However, patients with hypnotic usage and between 50 and 65 years of age had a higher increased risk of dementia (HR, 5.22; 95% CI, 2.62–10.41) than those older than 65 years (HR, 2.33; 95% CI, 1.90–2.88) (**Table 3**).

**Table 3:** Hazard ratios of dementia in patients with and without hypnotic usage stratified by age and sex.

Group	Hypnotic Users, No. (%) (N = 5693)		Hypnotic Nonusers, No. (%) (N = 28,465)		HR	(95% CI)	*P* Value
All	220/5473	(3.86)	424/28041	(1.49)	2.34	(1.92–2.85)	<.001
Sex							
Male	97/2423	(3.85)	183/12417	(1.45)	2.28	(1.68–3.10)	<.001
Female	123/3050	(3.88)	241/15624	(1.52)	2.39	(1.85–3.09)	<.001
Age							
50–65	27/2758	(0.97)	22/13903	(0.16)	5.22	(2.62–10.41)	<.001
>65	193/2715	(6.64)	402/14138	(2.76)	2.33	(1.90–2.88)	<.001

Abbreviations: CI, confidence interval; HR, hazard ratio.

All models are analyzed by Cox regression adjusted for hypertension, diabetes, hyperlipidemia, and stroke.

Both BZD users and non-BZD users had higher risks of dementia, but there were no significant differences between them (HR, 1.01; 95% CI, 0.76–1.33) (**Table 4**). No matter whether the patients used short-acting, intermediate-acting, or long-acting hypnotics, they appeared to be at greater risk of dementia than the no hypnotic usage group. A longer half-life for the hypnotic drug predicted a greater risk of dementia (HR, 1.65; 95% CI, 0.68–3.83) compared with the short-acting hypnotics, but it was not significant (*p*>0.05). Furthermore, no matter whether the subjects had been prescribed low doses, medium doses, or high doses, all appeared to be at greater risk of dementia. A higher prescribed dosage of hypnotics predicted a greater risk of dementia (HR, 1.53; 95% CI, 1.15–2.05) compared with the lower prescribed dosage.

**Table 4:** Hazard ratios of dementia in patients with and without hypnotic usage stratified by categories of hypnotics, half-lives of hypnotics and doses of hypnotics.

Group	Hypnotic Users, No. (%) (N = 5693)		Hypnotic Nonusers, No. (%) (N = 28,465)		HR	(95% CI)	*P* Value
Category							
BZD	76/1839	(3.97)	151/9409	(1.58)	1		
non-BZD	96/2716	(3.41)	190/13870	(1.35)	1.01	(0.76–1.33)	.351
Half-life							
Short-acting	159/4306	(3.70)	306/22019	(1.43)	1		
Intermediate-acting	39/856	(4.57)	76/4399	(1.79)	0.96	(0.61–1.52)	.523
Long-acting	15/318	(4.50)	19/1646	(1.16)	1.65	(0.68–3.83)	.098
Dose*							
Low	63/2655	(2.32)	192/13398	(1.41)	1		
Medium	103/2868	(3.47)	216/14639	(1.45)	1.07	(0.79–1.47)	.291
High	117/2605	(4.30)	208/13402	(1.53)	1.53	(1.15–2.05)	<.001

Abbreviations: BZD, benzodiazepine; CI, confidence interval; HR, hazard ratio.

All models are analyzed by Cox regression adjusted for hypertension, diabetes, hyperlipidemia, and stroke.

* Low: 7 to 30 defined daily dose (DDD) per year; medium: 31 to 90 DDD per year; high: at least 91 DDD per year.

In terms of the association of the diagnosis of dementia with the period of the last hypnotic prescription, current hypnotic users were associated with a significantly higher risk of dementia than remote hypnotic users (*p*<0.001; HR, 4.38; 95% CI, 3.03–6.35), and previous hypnotic users were associated with an increased risk of dementia compared to remote hypnotic users (HR, 3.44; 95% CI, 2.19–5.42) (**Table 5**). However, there were no significant differences between current hypnotic users and previous hypnotic users.

**Table 5:** Hazard ratios of dementia stratified by the period between the last hypnotic prescription and the dementia diagnosis.

Period*	Hypnotic Users, No. (%) (N = 5693)		HR	(95% CI)	*P* Value
Remote	65/3369	(1.89)	1		
Previous	46/746	(5.81)	3.44	(2.19–5.42)	<.001
Current	109/1358	(7.43)	4.38	(3.03–6.35)	<.001

Abbreviations: CI, confidence interval; HR, hazard ratio.

All models are analyzed by Cox regression adjusted for age, sex, hypertension, diabetes, hyperlipidemia, and stroke.

* Current: prescription of hypnotics ended within 30 days before dementia index date; previous: prescription ended 31 to 90 days before dementia index date; remote: prescription ended 91 days or more before dementia index date.

## Discussion

Our study found that patients with insomnia and long-term use of hypnotics have more than a 2-fold increased risk of dementia, despite excluding all patients with any diagnosis of SMI and PD. Patients with insomnia, regardless of whether they use BZD or non-BZD hypnotics, have higher risks of dementia. In addition, a long-acting half-life value and a higher prescribed dosage of hypnotics carry greater risks of dementia. It is suggested that the use of hypnotics, including both BZDs and non-BZDs, in patients with long-term insomnia, should be considered as a risk factor for dementia. Besides, patients with long-term insomnia and between the ages of 50 and 65 years have a higher risk of dementia than those older than 65 years. It is possible that older people as such have a higher risk of dementia, so the effect of hypnotics is not highly significant in this group.

### The Effect of Hypnotics

The increased risk of dementia in long-term insomnia patients that use hypnotics may be due to the effects of the hypnotics themselves. Other possible reasons are that sleep disturbance may be common in the prodromal phase of dementia [24] and dementia itself may produce sleep disturbances and circadian disruption by specific deterioration of the suprachiasmatic nucleus and the pineal gland [25], but other studies that focused on sleep disturbance and dementia did not support that view [26], [27]. They found that insomnia was not a predictor of dementia, but daytime sleepiness was. We found that those with more exposure to hypnotics had a higher risk of developing dementia. This result might be explained by the severity of insomnia or the effect of hypnotics in developing dementia. We could not make a clear distinction of the influence of developing dementia between insomnia and exposure to hypnotics in this study. However, the overall effect of insomnia and exposure to hypnotics in developing dementia is significant.

### The Association between Hypnotics and Cognitive Decline

Many studies have focused on the relationship between hypnotics and cognitive decline [28], [29], [30], [31], [32], [33], [34], [35], [36], [37], [38], [39], [40], [41], [42], [43]. An early study revealed the protective effects of BZDs on the development of dementia [1]. However, a recent meta-analysis has shown that the use of BZDs worsened cognitive function [28]. In our study, current and previous hypnotic users had an equal risk of developing dementia, and higher than that of remote users. This result clearly reveals that there is a higher risk of developing dementia after exposure to hypnotics without long-term discontinuation. A previous study using the LHID also suggested that the effect of BZDs decreased as the duration of discontinuation lengthened [3]. Therefore, early discontinuation of hypnotics might avoid the development of dementia.

### The Dose-response Relationship

Although we don’t know the relationship of the severity of insomnia and dementia, the dose-response relationship observed in our study between the dosage and the half-life values of hypnotics and dementia might be due to the accumulating adverse effects of BZDs. Block et al. reported that high diazepam doses affected retrieval and storage processes in selective reminding tasks, producing impairments qualitatively similar to those shown by demented patients; in contrast, low diazepam doses showed no impairment [29]. Hanlon et al. reported that current BZD use, especially at recommended or higher doses, was associated with worsened memory among community-dwelling elderly [44]. In our study, we also found an association between higher prescribed dosages of hypnotics and risks of dementia. In addition, patients using hypnotics with a long half-life had the highest risk of developing dementia. The results suggested that continuous exposure to BZDs may contribute to the development of cognitive impairment.

### The Pathophysiology of Cognitive Impairment

The pathophysiology of cognitive impairment after using BZDs is uncertain. The dynamic balance of the cholinergic and glutamatergic systems in the central nervous system, and the inhibitory actions of gamma-aminobutyric acid (GABA) signalizing via the GABA_A_ receptors are well known [45]. The increased GABAergic transmission associated with BZD opposes the deleterious effects of the neuroexcitotoxicity transmitter glutamate, which may be involved in the emergence of dementia [1]. Some studies have demonstrated that BZDs might hyperpolarize the neuron cell membrane and impair synaptic plasticity, which compromises the ability to form new memory [46]. Another possible mechanism is the down-regulation of the GABA receptors after long-term BZD exposure; this condition has been associated with cognitive impairment [47], [48], [49], [50]. We also found the use of non-BZD hypnotics carried a similar risk of developing dementia. This is reasonable because non-BZD hypnotics also signalize via GABA_A_ receptors, which might explain this condition.

### Strengths and Limitations

The strengths of this study in contrast to previous studies are that our data are based on a large and representative population-based sample. The size of the sample enabled a systematic examination of the effect across comorbidities and different covariates, and of the contribution of hypnotics. In addition, we examined the risks of dementia in terms of the different half-lives of hypnotics; this analysis has not appeared in other studies.

There are also some limitations to this study. First, because the LHID did not provide complete individual information, such as educational level, personal history of smoking and alcohol consumption, BMI, socioeconomic status, etc., all of which are known to contribute to dementia, we could not control all confounders. Also, we could not evaluate the severity of insomnia using the LHID. Second, the follow-up period may not have been long enough for patients to develop dementia in our study. Third, despite the large size of our sample, the number of cases in some categories was still relatively small, particularly for those from 50 to 65 years old and those using long-acting hypnotics. Last, insomnia might be caused by some mental illnesses, such as depression or anxiety disorders, which may have been underdiagnosed. We were not able to exclude those subjects with underdiagnosed mental illness in the current study setting.

### Conclusions

In conclusion, and based on our findings, we suggest giving careful consideration to prescribing BZD or non-BZD hypnotics to patients with long-term insomnia, especially those that are aged between 50 and 65 years. In addition, the lower the dosage and half-live values of the hypnotics used the better, because greater exposure to these medications leads to a higher risk of developing dementia.
